# Ion-Selective Electrodes for Measuring Potassium in Erythrocytes: a Model for Clinical Interpretation of the Results (a Pilot Study)

**DOI:** 10.17691/stm2022.14.3.05

**Published:** 2022-05-28

**Authors:** А.А. Astakhov, V.V. Kazartsev, K.V. Kuchkin, J. Barg

**Affiliations:** Head of the Department of Anesthesiology and Intensive Care Medicine; South Ural State Medical University, 64 Vorovskogo St., Chelyabinsk, 454092, Russia; Researcher; South Ural State Medical University, 64 Vorovskogo St., Chelyabinsk, 454092, Russia; Anesthesiologist; Central Clinical Hospital of the Presidential Administration of the Russian Federation, 15 Marshal Timoshenko St., Moscow, 121359, Russia; Anesthesiologist Former Senior Physician; Asklepios Academic City Hospital, 19 Brunnenallee, Bad Wildungen, 34537, Germany

**Keywords:** potassium in erythrocytes, potassium deficit, ion-selective electrodes, eryptosis, metabolic alkalosis

## Abstract

**The aim of the investigation** is to study the concentration of potassium in erythrocytes using the proposed method, potassium interconnection with other electrolyte and acid-base parameters of blood plasma, and to create the basis for clinical interpretation of the results.

**Materials and Methods:**

Potassium content in erythrocytes was measured using a blood gas analyzer with ion-selective electrodes in parallel with the laboratory procedure. Patients from intensive care units were randomly selected for the study.

**Results:**

No correlations of potassium with other plasma parameters have been found, however its buffer dependence on chlorine in plasma has been established. Minimal value of potassium concentration in erythrocytes (for 356 measurements) was 68.2 mmol/L, maximal — 210.2 mmol/L.

Following the logic of the acid-base status, a nomogram for clinical interpretation of intracellular potassium homeostasis has been developed. The low values are mainly connected with the deficit of potassium which is impossible to determine in blood plasma (e.g. in severe metabolic alkalosis or diuretic therapy). The elevated concentration of potassium in erythrocytes is caused by eryptosis: released potassium is absorbed by normal erythrocytes (protection from hyperkalaemia). So, the increased concentration of potassium indicates directly the presence of eryptosis triggers, i.e. inflammatory mediators, oxidative stress, and others, for example in sepsis. The results of the study have shown that measurement of potassium concentration in erythrocytes with the help of ion-selective electrodes is an effective method of monitoring its intracellular homeostasis. Potassium in erythrocytes is an independent biological marker which can provide clinically relevant information.

## Introduction

For decades, erythrocytes have been used as a cellular model for studying cell membranes. The development of highly technological methods in molecular biology (including proteomics) in the last 15–20 years has led to the idea that erythrocytes are an organ which is involved in many “noncanonical” functions (other than gas transport) which impact systemic metabolic homeostasis [1].

Erythrocytes are rather poorly adaptable for survival. They do not contain any nuclei or organelles and are not capable of protein synthesis and regeneration. Due to the absence of mitochondria, only anaerobic glycolysis is possible in erythrocytes, i.e. energy depletion in these blood cells occurs faster — the process is called “erythrocyte hypoxia” [2].

Erythrocytes are very sensible and vulnerable cells with a highly specialized and organized membrane structure. They are exposed to the action of metabolites and mediators in various tissues and are constantly transported through the stress zones [3].

An oxidative stress in the blood circulation can damage erythrocytes. If there is a threat to their membrane destruction, eryptosis takes place: programmed death of erythrocytes occurring before they reach an average lifetime (100–120 days). An important physiological function of eryptosis is to prevent hemolysis and the resulting complications. Numerous triggers of eryptosis have been discovered including hyperosmotic shock, energy depletion, oxidative stress, inflammatory mediators, oxygen free radicals, hepatic failure, heart failure, sepsis, fever, dehydration, and antibiotics [3]. Eryptosis process is a well-studied process; the intracellular mechanism always follows one and the same sequence irrespective of the trigger origin [3, 4].

It is known that 98% of the total amount of potassium in the organism is inside the cells; however, there has been no suitable method of assessing internal potassium homeostasis so far.

Flame photometry and atomic spectrometry are the current standards for measuring cations in erythrocytes. However, both of these methods are technically complicated and unsuitable for clinical application.

The first attempts to measure erythrocyte potassium content with the help of ion-selective electrodes were described in 1978 [5], but the technique did not receive further development. A simple method has been developed by us which makes it possible to measure the erythrocyte potassium content with ion-selective electrodes on any blood gas analyzer in several minutes [6]. This method is based on the process of blood preparation for flame photometry: a blood specimen is first subject to hemolysis and then the potassium concentration is determined on a standard blood gas analyzer with subsequent calculations (instructions for the work according to this method may be requested from the authors). The method is validated by means of flame photometry. The normal control range of potassium in erythrocytes (35 healthy subjects) according to our data is 90−110 mmol/L. The real concentration of erythrocyte potassium is higher and is reported to be 148.0±2.0 mmol/L (measurements were done using nuclear magnetic resonance) [7]. In compliance with the law of electrochemical neutrality, the intracellular concentration of potassium in other tissues must largely correspond to the concentration of sodium in plasma. The lower values of erythrocytes obtained by us by means of flame photometry may be explained exclusively by dilution (during centrifuging, erythrocytes never reach the same cell density as in other tissues). So, we measure the potassium content in somewhat “diluted” tissue.

**The aim of the study** is to assess the efficacy of measuring potassium concentration in erythrocytes in clinical practice using ion-selective electrodes and to study its interconnection with other parameters of blood plasma such as electrolytes and acid-base balance.

## Materials and Methods

Patients (192 men and 178 women aged from 39 to 82 years) were randomly selected from three interdisciplinary intensive care units irrespective of their sex, age, diagnosis, or clinical state. The “typical population” of intensive care units is presented by patients with severe surgical infections and sepsis, postoperative complications and polyorgan insufficiency. There were 370 measurements performed from October 2018 to March 2020. Blood samples from the arterial or central venous line were collected with a standard syringe with heparin. The potassium content in erythrocytes was measured using two analyzers: ABL90 FLEX (Radiometer, Netherlands) and cobas b 221 (Roche Diagnostics GmbH, Germany) in parallel with usual analyses.

The study complies with the Declaration of Helsinki (2013) and was approved by the Ethics Committee of the South Ural State Medical University (Chelyabinsk, Russia). Written informed consent was obtained from all patients.

The erythrocyte potassium concentration (K_er_) was calculated in the following way:

Кall =Кhem ⋅12; Кpl.d=[Кpl⋅(100−Ht)]/100; Кer.d=Кall −Кpl.d;Кer =Кer.d⋅100/Ht,

where К_hem_ is a measured potassium concentration in hemolysate; К_all_ — a total amount of potassium in hemolysate; К_pl.d_ — proportion of plasma potassium in hemolysate; K_er.d_ — proportion of erythrocytic potassium in hemolysate; K_pl_ — proportion of plasma potassium in the normal sample; K_er_ — potassium concentration in erythrocytes (mmol/L). The samples were diluted in 1:12 ratio.

The total deficit of potassium is calculated in the following way:

BMI⋅0.65⋅(90−Ker),

where 0.65 is a cell mass; 90 is a lower limit of normal (or target result).

**Statistical analysis** was performed using the SPSS IBM (v. 23) software package. The data sample (various patients) is unrelated. The data do not follow completely the normal distribution (left shift), which is typical for many physiological parameters. The lowest registered value for potassium content in erythrocytes was equal to 50.4 mmol/L, the highest was 240.0 mmol/L. Fourteen extreme values (<60 and >210 mmol/L) were defined as spikes and excluded from the analysis. The rest 356 measurements have been statistically processed. The correlation analysis was done calculating the Spearman’s correlation coefficient. The data were presented as М±σ and Ме [Q1; Q3].

## Results and Discussion

**Figure 1.**
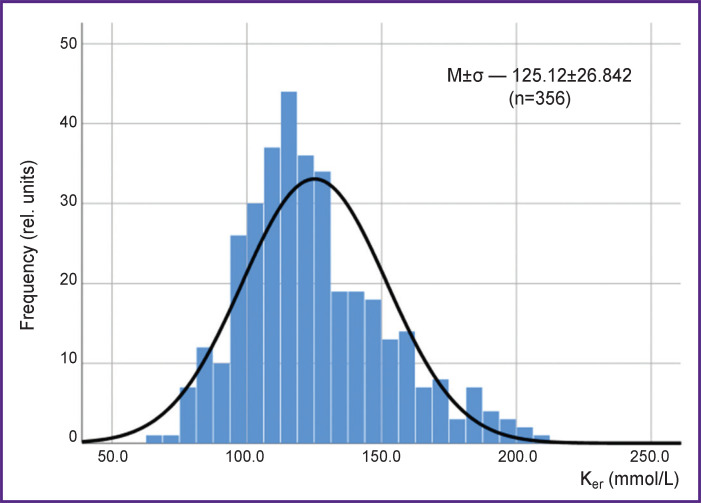
Histogram of data distribution

The minimal value of erythrocyte potassium concentration for 356 measurements made up 68.2 mmol/L, maximal — 210.2 mmol/L (Figure 1); М±σ — 125.1±26.8 mmol/L; Ме [Q1; Q3] — 20.5 [106.5; 140.9] mmol/L.

Plasma potassium and other parameters of electrolyte and acid-base balance also had deviations from the normal distribution to various degrees (data are not presented). No correlations between potassium and other parameters of electrolytes and acid-base status have been detected by us. This suggests that intracellular potassium has rather complex relations of the buffer character with electrolyte and acid-base parameters of plasma and most likely with the main plasma anion, chlorine. We introduced three derived parameters, the metabolic equivalents (analogs) of which are the parameters of the acid-base balance, all of them referring to chlorine in plasma. Potassium concentration in erythrocytes may be considered a metabolic equivalent of pCO_2_.

1. K_er.st_ is erythrocyte potassium, “standard value”. This parameter corresponds theoretically to the “normal” ionic balance with chlorine (about 100 mmol/L), consequently, K_er.st_=Cl. Its analog is standard bicarbonate, SB (pH 7.4; pCO_2_=40 mm Hg; HCO_2_=24 mmol/L).
2. K_er.exc_=K_er_–K_er.st_ — excess of potassium in erythrocytes (the difference between the measured and standardized value). Analog is the base excess (BE). The value of this parameter may be positive or negative depending on the appropriate chlorine concentration. It is important to take into consideration that values +/– change places since potassium is a cation. Thus, elevated K_er.exc_ denotes theoretically intracellular metabolic acidosis and vice versa.
3. The ratio pK_er_=Cl/K_er_ is similar to рН in acid-base balance (pH=pCO_2_/HCO_2_). To make the interpretation more conventional, we have swapped cations and anions. Since the calculated values are too small (norm ≈1.0), they are multiplied by 7 for convenience. Thus, values greater than 7 denote intercellular alkalosis, those smaller than 7 speak of intracellular acidosis.

**Figure 2.**
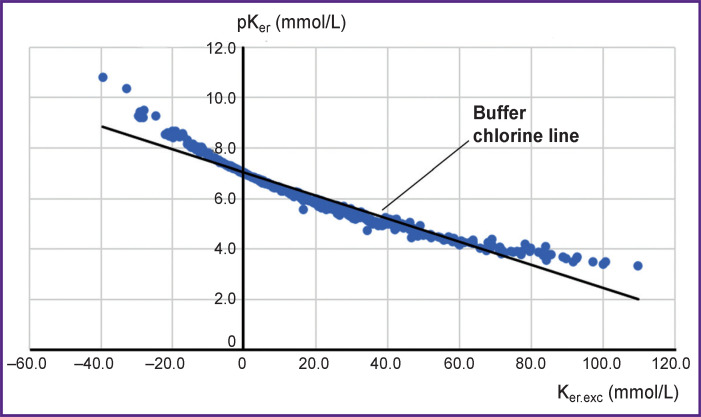
Distribution of derivatives of K_er_ parameters

The graphical presentation of K_er.exc_ and pK_er_ (Figure 2) confirms our hypothesis about a close buffer connection of the potassium concentration in erythrocytes (the main cellular cation) with the concentration of chlorine in blood plasma (the main extracellular anion). The Spearman’s correlation coefficient between K_er.exc_ and pK_er_ was –0.998 (p<0.001).

**Figure 3.**
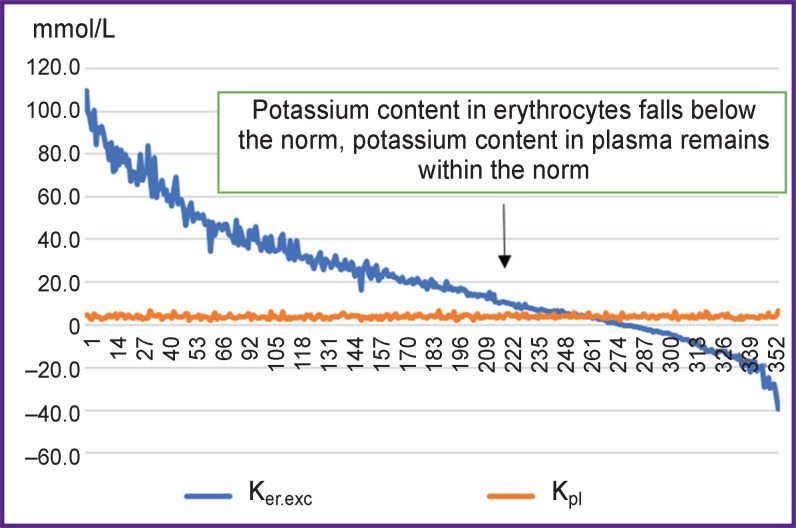
Distribution of K_er.exc_ parameter and plasma potassium (K_pl_) for 352 measurements

The graphical presentation of the erythrocyte potassium values and the potassium concentration in plasma clearly demonstrates absence of any dependence. At great fluctuations of potassium concentration in erythrocytes (up to the critical values), potassium concentration in plasma changes insignificantly (Figure 3).

The curve of erythrocyte potassium concentration has an exponential shape typical for numerous physiological processes (as, for example, the hemoglobin saturation curve, pressure/ volume curve in the lungs, etc.).

The linear correlation confirms our assumption that potassium concentration in erythrocytes has a close buffer dependence on chlorine in plasma since all measurements are located practically on the same line (see Figure 2), i.e. chlorine buffer line. This line is very like the buffer line in the Siggard–Andersen acid-base nomogram. No correlation is also observed between the derivatives of the parameters introduced by us and plasma potassium.

### Potassium content in erythrocytes is below the norm (<90 mmol/L)

Three main causes of the low potassium concentration in erythrocytes may be assumed: potassium loss (acute or chronic); ATPase inhibition (Na/K pump) without potassium loss; acute osmotic effect of sodium and water — “sodium attack” as in acute renal failure. The combination of the above causes is also possible. Complete absence of correlation between potassium content in plasma and erythrocytes leads to the conclusion that potassium content in plasma cannot reflect its deficit in erythrocytes and that erythrocyte potassium is not involved in regulation of the potassium level in plasma.

Potassium deficit in humans arises due to traumas, shortage of food and water. There is a protective mechanism against these factors: retention of sodium and water (hyperaldosteronism). If plasma potassium concentration increases, it is immediately excreted by the kidneys. Besides, erythrocytes may be the first cells to lose potassium.

### Potassium content in erythrocytes is increased (>110 mmol/L)

The increased content of potassium in erythrocytes has not yet been described. To understand the reason of this event, let us recollect eryptosis. In the final phase of eryptosis, KCl and water are fully excreted from erythrocytes to plasma. The dead erythrocytes are eliminated from the blood circulation, while released KCl remains in plasma [8]. Erythrocytes represent a potassium concentrate, its concentration in erythrocytes is 30 times higher than in plasma [9]. So, if anemia associated with eryptosis leads to the decrease of the Hb level from 13 to 12 g/L, then the released potassium must increase the potassium concentration in plasma from 4.5 to fatal 9.5 mmol/L, but it does not occur since the released KCl is immediately absorbed by healthy erythrocytes. This process is a physiologically logical continuation of the protective function of eryptosis first against hemolysis and then against hyperkaliemia. This results in the elevation of potassium concentration in erythrocytes. It is proved to be correct by statistically significant negative correlation between potassium in erythrocytes and hematocrit (hemoglobin) which may be accounted for by elevated eryptosis.

Excess potassium may be easily eliminated by the kidneys but erythrocytes act as an independent organ and “solve the problem” at the organ level. Chlorine absorption by erythrocytes is confirmed by the decrease of chlorine in blood plasma at K_er_ more than 120 mmol/L (statistical data are not given). One should remember the proved inability of chlorine ions to move by osmotic gradient (into the cell) as it takes place with cations of potassium and sodium [9]. In other words, decrease in potassium concentration may be explained by its absorption by erythrocytes.

**Figure 4.**
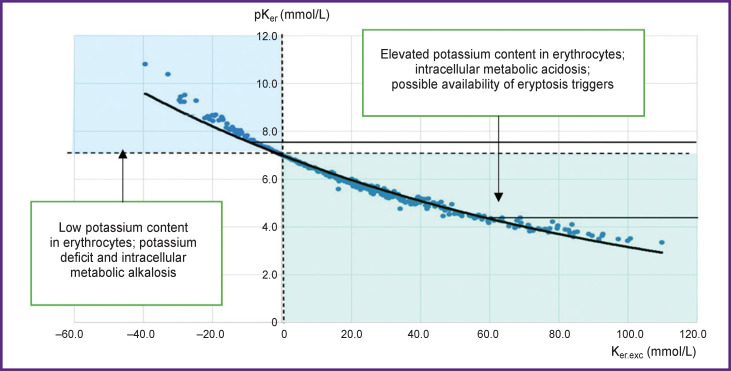
Nomogram for clinical interpretation of potassium content in erythrocytes Two dash lines correspond to the normal value of pKer (≈7) and Ker.exc (≈0). The exponential diagonal chlorine curve divides the nomogram into two zones which reflect all possible measured values

This inevitably leads to acidosis (as in plasma) therefore we may speak here about theoretical intracellular acidosis. And on the contrary, if potassium loss occurs, theoretical intracellular alkalosis may be suggested. Distinct linear relations between the parameters allow us to create a nomogram for interpretation of the measurement results (Figure 4). The majority of the measurements are in the zone of cellular acidosis (to the right of the zero line). There are considerably fewer measurements in the zone of potassium deficit and cellular alkalosis and the linear segment of the nomogram (compensation zone) is much shorter. The data obtained confirm a well-known fact that metabolic acidosis is the most frequent shift in the acid-base balance of the body and human physiology is much more “specialized” in compensation of acidosis and less in compensation of alkalosis. This also explains the asymmetry of the normal distribution histogram — the left shift.

**Figure 5.**
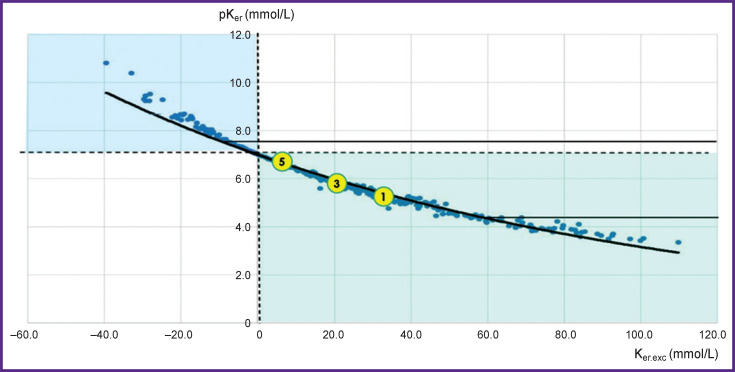
Potassium content in erythrocytes in patient with peritonitis (67 years) Measurements were performed on days 1, 3, 5 in the intensive care unit

As the increase of potassium content in erythrocytes is directly related to the eryptosis intensity, the elevated potassium concentration in erythrocytes is most likely to reflect general aggressivity/toxicity of the intracellular fluid. Massive eryptosis is known to occur in sepsis [10], therefore, the increase of potassium content in erythrocytes may reflect sepsis severity. Hypocalcemia observed in sepsis is also indirect evidence of calcium movement into erythrocytes at the initial stage of eryptosis. The data obtained by us prove this supposition: in clinical improvement, K_er_ values drop (Figure 5).

Complete absence of correlation between the potassium content in erythrocytes and other parameters of blood plasma confirms that erythrocytes are a separate organ. And consequently, these cells possess a morphological and functional sovereignty, self-regulation system, etc. At the same time, erythrocytes, being a liquid organ, are dynamically distributed between all organs and tissues and are actively involved in the general homeostasis of the body. Plasma is an extracellular space of erythrocytes which needs exploration for the diagnosis of their functional state. This approach agrees with the traditional interpretation of other laboratory parameters: hepatic enzymes are determined to assess the liver function; creatinine is measured to assess the kidney function.

Erythrocytes are very sensible and vulnerable cells; therefore, K_er_ increase may be an early prognostic sign of polyorgan insufficiency.

Taking into consideration a large number of potential triggers of eryptosis [4], it may be assumed that practically any pathophysiological escalation may be accompanied by elevated eryptosis and increased potassium concentration in erythrocytes.

**Figure 6.**
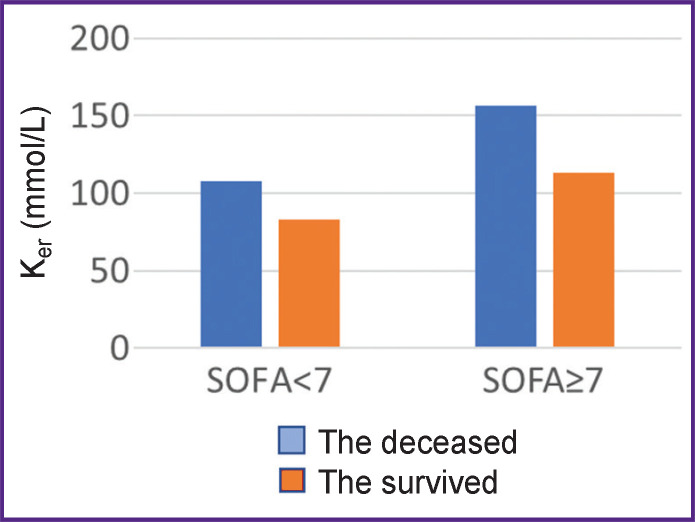
Relation between the K_er_ concentration, measured on day 1 in the intensive care unit, and the lethal outcome

Values K_er_ have been established to comply with the SOFA scale (Figure 6).

**Figure 7.**
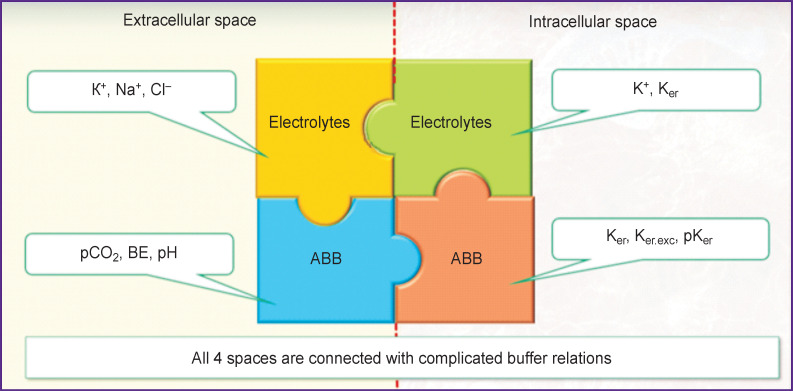
The model of electrolyte and acid-base balance (ABB) BE is base excess

Evidently, there are very close and mostly unexplored interconnections between extra- and intercellular electrolyte and acid-base balance. They may be presented graphically as a model (Figure 7).

The clinical cases presented below serve to illustrate this model.

### Case 1: acute potassium deficit with metabolic alkalosis

*Marked intestinal atony (up to compartment syndrome) with a severe metabolic alkalosis developed in 70-year-old patient in the postoperative period (replacement of the abdominal aorta). BE was +22, plasma potassium — 4.2 mmol/L (corrected hypokalemia), erythrocyte potassium — 65 mmol/L, potassium deficit — 1365 mmol*.

*This case, severe metabolic alkalosis, is difficult to treat. Its special resistance to therapy is explained, in our opinion, just by the combination of intra- and extracellular metabolic alkalosis to which a human organism is not evolutionarily adapted. The only effective therapy was a forced replacement therapy with potassium preparations (even after the achievement of normokalemia) under the control of its content in erythrocytes. The patient received daily 350–400 mmol of potassium for 2 days (in normokalemia!). On day 1, potassium content increased to 85 mmol/L, on day 2 to 98 mmol/L, BE fell to +8. Significant clinical improvement with normalization of intestinal peristalsis was observed*.

### Case 2: chronic potassium deficit during diuretic therapy

*A 55-year-old patient suffered from Wolff–Parkinson–White syndrome, paroxysmal supraventricular tachycardia, and 180–200-bpm heart rate. Cardioversion was indicated after 8 h of resistance to therapy with the signs of heart failure. Plasma potassium was 3.7 mmol/L, erythrocyte potassium — 68 mmol/L, potassium deficit — 1200 mmol. During preparation to a short-term anesthesia, infusion was quickly started (10% glucose + 20 mmol/L KCl, 20 mmol Mg^2+^ and 24 units of insulin)*

*Five minutes later, there occurred a spontaneous cardioversion to sinus rhythm, and electrical cardioversion was not necessary*.

*We often observe a low level of potassium in erythrocytes in patients receiving diuretic therapy. It is highly likely that many of them have unrecognized potassium deficit which may potentially result in dangerous cardiac arrhythmias*.

### Case 3: acute depression of a sodium-potassium pump

*A 55-year-old patient with an excessive weight (140 kg) underwent non-invasive lung ventilation because of acute respiratory insufficiency with decompensated respiratory acidosis (pCO_2_ — 130 mmol/L), plasma potassium — 5.6 mmol/L, and erythrocyte potassium — 55 mmol/L*.

*No special therapy was conducted. During 2 days after the improvement of the respiratory situation, pCO_2_ was 60 mm Hg and potassium in erythrocytes was within the norm — 92 mmol/L*.

## Conclusion

The results of our study allow us to make the following conclusions:

1. Measurement of potassium concentration in erythrocytes using ion-selective electrodes is a promising method of monitoring intracellular potassium homeostasis. Automation of the proposed method will promote its implementation in routine clinical practice.
2. Erythrocytes may be considered a separate organ actively participating (except for gas exchange) in general homeostasis of the organism. Intracellular potassium homeostasis is not directly connected with regulation of plasma potassium. The intracellular potassium concentration is an independent biological marker which has a close buffer connection with plasma chlorine.
3. The model of electrolyte and acid-base balance may serve as an initial base for clinical interpretation and study of the diagnostic potential of erythrocyte potassium.
4. A low potassium concentration may be an early sign of its cellular deficit which is impossible to determine by potassium concentration in plasma. An elevated potassium concentration in erythrocytes indicates intensive eryptosis (availability of its triggers), may be indirect evidence of the general aggressivity/toxicity of the extracellular fluid or severity of inflammation and supplement thereby the criteria of Sepsis-3 concept.
